# Breast Cancer During Pregnancy

**DOI:** 10.7759/cureus.2941

**Published:** 2018-07-08

**Authors:** Sajid Durrani, Shomaila Akbar, Humariya Heena

**Affiliations:** 1 Medical Oncology, King Fahad Medical City, Riyadh, SAU; 2 Radiation Oncology, King Fahad Medical City, Riyadh, SAU; 3 Research Center, King Fahad Medical City, Riyadh, SAU

**Keywords:** breast cancer, pregnancy, breast cancer treatment, breast cancer diagnosis

## Abstract

Breast cancer remains the most common cancer in women. A diagnosis of cancer during pregnancy is uncommon. In recent decades, obstetricians are seeing an increasing number of women who become pregnant or desire to become pregnant after breast cancer treatment because of a delay in childbearing for a variety of reasons, including cultural, educational, and professional. Consequently, breast cancer in young women often occurs before the completion of reproductive plans.

A discussion among the patient, the oncologist, and the obstetrician on the relative benefits of early delivery followed by treatment versus commencement of therapy while continuing the pregnancy is of utmost importance in order to reach a consensual decision. The best available evidence suggests that pregnancy after breast cancer increases the risk of recurrence. The birth outcome in women with a history of breast cancer is no different from that in the normal female population; however, increased risks of delivery complications have been reported in the literature.

As concurrent pregnancy and breast cancer are uncommon, there are no data from large randomized trials; hence, recommendations are mainly based on retrospective studies.

## Introduction and background

The incidence of breast cancer in pregnancy is approximately 1 in 3,000 and can reach up to 3% [1]. The prevalence of pregnancy-associated breast cancer may be increasing owing to delayed childbearing, and despite its low incidence, breast cancer is the second most common cancer in pregnant women. In this review, we aimed to provide insights into the available diagnostic and therapeutic modalities for breast cancer during pregnancy and to elaborate on the prognosis and best practices for these concurrent conditions.

A case series conducted at Memorial Sloan-Kettering Cancer Center (New York, NY, USA) in 1991 revealed that 44 of 56 patients with breast cancer did not have cancer diagnosed until after delivery, although more than half of the women already had symptoms prenatally. It was observed that the women frequently presented with tumors having adverse pathological prognostic features (median tumor size, 3.5 cm), suggesting either that the breast masses were not clinically recognized or that follow-up was delayed [2]. Moreover, many masses discovered during pregnancy and lactation are attributed to physiologic changes and likely to be discounted by the patient.

The available data on pregnant patients with breast cancer are primarily from either case reports or case-control studies because of the ethical restrictions in conducting randomized clinical trials in such patients. Pregnant women with breast cancer are more likely to have larger tumors, positive nodes, metastases, and vascular invasion. In a retrospective case-control study, Zemlickis et al. found that breast cancer during pregnancy was more likely to be diagnosed at a later stage when distant metastasis had already occurred [3].

Bonnier et al. conducted an extensive study on hormone receptor status in 75 pregnant patients with breast cancer and a control group of 182 non-pregnant patients with breast cancer. Of the pregnant women, 56% to 83% had positive nodes at diagnosis versus 38% to 54% of the non-pregnant women. In the pregnant group, 42% of cancers were estrogen receptor (ER)-negative and progesterone receptor (PR)-negative, compared with 21% in the age-matched controls [4].

Many studies support the theory that ER-positive status is decreased in pregnant patients with breast cancer owing to receptor downregulation in pregnancy. Costa et al. hypothesized that high levels of circulating estrogens might even prevent ER-positive cancers [5].

## Review

Risk of breast cancer

The medical literature has already established that nulliparous women have a higher risk of developing breast cancer than multiparous women [6]. Similarly, nulliparous women having their first child after 30 years of age are at a slightly higher risk of developing breast cancer than women who are usually exposed to higher estrogen levels over a prolonged period. Early menarche (before the age of 12 years) and menopause after the age of 55 years are also significant risk factors. Cyclical hormone levels also predispose women to breast cancer over time. This may be because breast cells grow and divide in response to hormones, such as estrogen, and pregnancy leads to an interruption of the normal cyclical hormone levels [7].

Diagnosis and presentation

Breast cancer in pregnancy most often presents as a painless mass or thickening in the breast occasionally associated with discharge from the nipple. About 95% of women in one study and 82% of women in another study presented with a painless mass [8]. Normally, the mean breast weight doubles in pregnancy from 200 g to 400 g, resulting in increased firmness and density of the breast, which make the interpretation of the clinical examination and mammogram results more difficult. The hypertrophy and engorgement of the breasts may make both physical examination and mammographic imaging more challenging in pregnant than in non-pregnant patients. The preliminary investigation may be performed in non-pregnant women; however, for pregnant women, particular attention should be paid to the risks of ionizing radiation exposure for the fetus.

*Physical Examination* 

All pregnant women should undergo clinical breast evaluation as part of the first physical examination. If a woman presents with a breast lump during pregnancy, she should be referred to a team of breast specialists.

Mammography

Although mammography with abdominal shielding can be performed with minimal risk during pregnancy, mammograms are difficult to interpret and hence, not advisable for the investigation of breast cancer in pregnant women [4]. A mammogram during pregnancy may reveal calcification, asymmetric density, axillary lymphadenopathy, and skin and trabecular thickening, which are helpful in the diagnosis of pregnancy-associated breast cancer [5].

Ultrasonography

Ultrasonography is a sensitive (100% sensitivity in one series) investigation method in pregnant and lactating women based on three small series [8]. The most common sonographic finding is an irregular mass lesion with posterior acoustic enhancement. A marked cystic component is also seen occasionally. Ultrasonography is useful in detecting axillary metastases and in assessing response to systemic treatment in pregnant women with breast cancer [9]. Therefore, ultrasonography is the preferred imaging modality because of the radiodensity of the breast in the pregnant or lactating state. Ultrasonography can also aid in the assessment of the response to neoadjuvant chemotherapy. Hence, it is a simple and sensitive modality for the initial evaluation of breast cancer in pregnant and lactating women.

Chest Radiography 

With adequate shielding, chest radiography is considered safe during pregnancy, as the expected fetal dose is less than the harmful threshold [10]. However, its use is recommended only when exclusively indicated.

Magnetic Resonance Imaging (MRI)

MRI will most often be the procedure of choice because it does not use ionizing radiation and can identify bone metastasis [11]; however, MRI is associated with risks to the fetus due to heating and cavitation [12]. Although some radiologists remain opposed to the use of MRI in the first trimester, there is currently no consensus on this matter. A United Kingdom (UK) medical device agency recommends that MRI should be avoided during the first trimester, as there is a theoretical risk of fetal damage due to the high magnetic field [13].

As the contrast agent (gadolinium) can cross the placenta and its effects on the developing fetus are unknown, its use should be avoided. Gadolinium is classified by the Food and Drug Administration (FDA) as a Pregnancy Category C drug and is associated with fetal anomalies [14]. Positron emission-computed tomography, computed tomography, and pelvic radiography involve more radiation than MRI, and hence are not the preferred imaging modalities [15-17]. Bone scans, although rarely used, result in only 0.00194 Gy of radiation exposure to the fetus. As a consensus in clinical practice, all staging investigations that are likely to cause any risk to the fetus should be done only where a positive result would alter immediate management (Level IV).

Pathology

Because normal breast tissue shows atypical cytomorphologic features during pregnancy and lactation, evaluation of aspiration samples is difficult [18-21]. Core needle biopsy may be an appropriate initial procedure with a sensitivity of 90%; however, it increases the risk of milk fistula formation, infection, and bleeding [20-22].

Biopsy of a suspicious mass has been accepted as the gold standard for the diagnosis of breast cancer [23]. Appropriate risk reduction strategies include the use of prophylactic antibiotics and meticulous attention to hemostasis.

Principles of treatment

Treatment during pregnancy requires a discussion among the pregnant woman with breast cancer, the oncologist, and the obstetrician, on the relative benefits of early delivery followed by treatment versus commencement of therapy while continuing the pregnancy. Another factor preventing the administration of optimal therapy for the mother with breast cancer is the concern for the well-being of the fetus. In the selection of treatment, the factors considered at the time of diagnosis are the current stage of disease, hormone receptor status, and trimester of pregnancy. There should be a discussion about pregnancy and future fertility, and the patient should be informed that treatment for breast cancer may contribute to or cause infertility. The American Society of Clinical Oncology guideline highly recommends that an oncologist, along with a fertility specialist, counsel the patient about fertility preservation. Embryo and oocyte cryopreservation are both standard fertility procedures in most advanced centers worldwide, although their use is limited in developing countries.

Surgery

(1) First trimester: Surgery with mastectomy and axillary staging is recommended in the first trimester of pregnancy. Breast-conserving surgery (BCS) is not preferred in the first trimester because this technique requires radiation therapy, and radiation therapy has to be delayed during the first trimester. Breast reconstruction is also not an option in the first trimester; it should be delayed to limit the anesthetic exposure of the patient and the fetus as much as possible, as most patients require general anesthesia. Hence, pregnant women undergoing surgery under general anesthesia are more likely to give birth to low birth weight infants owing to prematurity and intrauterine growth retardation. Surgery is delayed up to the 12th week of gestation to avoid the high risk of spontaneous abortion, and the fetus is monitored according to gestational age. A study was conducted on 720,000 pregnant women among whom 5,405 underwent surgery, and it was found that there was an increased risk of neonatal mortality but no increase in congenital malformations and stillbirths [24]. Thus, surgery can be safely performed in patients with pregnancy-associated breast cancer with no unexpected complications.

(2) Second and third trimesters: Most of the patients in the published literature underwent mastectomy with axillary clearance. BCS with axillary dissection may be possible, especially in women in whom cancer is diagnosed in the second or third trimester; however, in women with advanced disease at presentation, neoadjuvant chemotherapy and surgery should be performed later in pregnancy or postpartum. The effect of sentinel node biopsy (SNB) has not been systematically evaluated in pregnant patients with breast cancer. SNB with isosulfan blue dye is not recommended for pregnant women, as anaphylaxis has been observed with the use of blue dye [25]. SNB with radioisotopes has been estimated to be associated with low radiation exposure to the fetus. Some have offered SNB after counseling the patient about its efficacy and safety because nodal metastases are commonly found in pregnancy-associated breast cancers and nodal status affects the choice of adjuvant chemotherapy [25]. The risks of SNB in pregnant women are unknown. Although some studies have reported the safety of SNB, data supporting this assertion are insufficient. For patients in whom cancer was diagnosed in the late second and third trimesters, lumpectomy and axillary dissection followed by irradiation after delivery are preferred over axillary dissection [26-29]. If the woman is far from term, BCS can be followed by chemotherapy after the first trimester and irradiation can be added after delivery. Modified BCS for Stage I and II tumors do not seem to have an advantage over modified radical mastectomy in terms of survival.

The common effects of pregnancy and cancer include dyspnea, nausea, and vomiting. The same treatment protocol is followed for these symptoms irrespective of the association with cancer or pregnancy. Factors that make anesthesia use difficult in pregnant patients include the hypercoagulable state, delayed gastric emptying, decreased functional residual capacity of the lungs, and increased blood volume and cardiac output. Therefore, adequate preoxygenation, rapid sequence induction with cricoid pressure, and other additional measures should be taken to prevent complications.

Radiotherapy

As any exposure to radiation may put the fetus at risk, adjuvant radiotherapy is usually delayed until after delivery.

Chemotherapy

(1) Maternal effects of chemotherapy: Physiological changes in pregnancy may alter the pharmacokinetic and pharmacodynamics of chemotherapy. These changes include alterations in hepatic metabolism, renal plasma flow, and plasma protein binding, all of which may affect drug clearance [30]. Amniotic fluid may act as a pharmacological third space and may delay the elimination of agents, such as methotrexate, resulting in increased toxicity.

(2) Fetal effects of chemotherapy: All drugs have the potential to cross the placenta depending on their physical and chemical properties. Chemotherapy can have several long-term effects, including gonadal dysfunction, germ-cell mutagenesis, teratogenicity in subsequent generations, and impaired physical and neurologic development.

(3) First trimester: When chemotherapy is given during the first trimester, there is a significant risk of spontaneous abortion and up to a 17% estimated risk of fetal malformations [10]. Hence, chemotherapy is avoided during this stage of pregnancy.

(4) Second and third trimesters: As organogenesis is complete at these stages, chemotherapy is widely used in the second and third trimesters. The only prospective series on this issue showed that there were no detectable congenital abnormalities and no maternal or fetal deaths in 24 pregnant women were treated with FAC (5-fluorouracil, Adriamycin, and cyclophosphamide) [9]. In another study of 28 patients who were treated with three different regimens (doxorubicin and cyclophosphamide; epirubicin and cyclophosphamide; and cyclophosphamide, methotrexate, and 5-fluorouracil), there were no deaths or congenital malformations when chemotherapy was given after the first trimester [10].

Although the spontaneous onset of labor and preeclampsia have been reported in patients who received chemotherapy, it is not known whether the incidence of these complications is higher than that in the healthy population, or to what extent chemotherapy affects the underlying disease. Myelosuppression occurring around the time of delivery may put the baby and mother at risk of developing sepsis and hemorrhage; hence, it is recommended that chemotherapy should be avoided at least three weeks before delivery so that maternal blood counts are optimal. Transient leukopenia without infectious sequelae has been reported in neonates born to mothers receiving chemotherapy. Children exposed to chemotherapy in utero are expected to have a higher risk of intrauterine growth retardation; however, the exact incidence is unknown [31-37].

Breastfeeding

Some studies have suggested that breastfeeding may slightly lower breast cancer risk, especially if breastfeeding is continued for one and a half to two years or if several children are breastfed [38]. A possible reason for this may be that both pregnancy and breastfeeding reduce a woman’s total number of lifetime menstrual cycles. However, as not all studies support this notion, more research is needed in this area.

Chemotherapeutic agents and toxicities

Pregnancy is a state of increased plasma volume, decreased albumin concentration, decreased gastric motility, and the possible existence of the amniotic sac as a third space. Almost all chemotherapy agents cross the placenta. Therefore, it is reasonable to avoid or reduce chemotherapy drugs for one month before delivery to prevent undue pressure on the newborn's liver and kidneys, which are not able to metabolize or excrete those drugs quickly. Nevertheless, after delivery, cyclophosphamide, methotrexate, and doxorubicin should be avoided as they can enter breast milk.

A French survey found that among women receiving breast cancer chemotherapy 25.2 days before delivery, the incidence of leukopenia was relatively high in their newborns [40]. In a prospective clinical trial, 24 patients were administered fluorouracil, cyclophosphamide, and doxorubicin in the second and third trimesters similar to non-pregnant patients, except in the first trimester [9].

Anthracyclines

Anthracycline-based chemotherapy seems to be safe during the second and third trimesters. Based on a review of 160 patients who took anthracyclines during pregnancy, Germann et al. suggested that a < 70 mg/m^2^ dose of doxorubicin per cycle should be given as a short infusion or bolus. The risk of fetal toxicity was increased 30-fold when the dose of doxorubicin per cycle exceeded 70 mg/m^2^ (P = 0.037) [29]. Maternal exposure to anthracyclines is another long-term risk factor for fetal cardiotoxicity. However, in a comprehensive published review, no case of fetal cardiotoxicity was seen [40].

Taxanes

Paclitaxel has been used in combination with cisplatin from 28 weeks of pregnancy and in combination with epirubicin from 14 weeks with no fetal or maternal complications [41]. Similarly, docetaxel has also been used both as a single agent and in combination with doxorubicin after 14 weeks of pregnancy in patients with breast cancer with no specific complications [33]. Concerning other antineoplastic agents, vinorelbine in combination with 5-fluorouracil has been given to three patients in the second and third trimesters with no adverse effects [34].

Cyclophosphamide

Cyclophosphamide has been known to cause frequent congenital malformations. The relative risk of congenital disabilities with alkylating agents has been found to be ranging from 4% to 13% in late pregnancy [42]. However, it is difficult to distinguish the effects of any one drug, as the patient is exposed to multiple modalities of treatment.

Methotrexate

Methotrexate is strongly contraindicated because it is a known abortifacient and the leading cause of chemotherapy-related birth defects.

Fluorouracil

The teratogenicity of fluorouracil remains largely unclear. It has been associated with bony aplasia and hypoplasia, although concurrent exposure to at least 5 rads (0.05 Gy) during diagnostic testing cannot be discounted.

Antiemetics

There is a significant association between corticosteroid use in the first trimester and cleft palate. However, corticosteroids as part of the antiemetic regimen seem to be safe during and after the second trimester. The use of ondansetron during the first trimester does not seem to cause malformations [35-37]. Hence, antiemetic regimens with corticosteroids and ondansetron may be safe during the second and third trimesters of pregnancy.

Granulocyte Colony-stimulating Factor (G-CSF) and Erythropoietin

G-CSF and erythropoietin have been used safely in pregnant patients.

Trastuzumab

Human epidermal growth factor receptor-2 (HER2) expression is high in embryonic tissues, suggesting that it has a role in embryonic development, and placental transfer of trastuzumab has been observed in animal studies. In a previous study, three patients received trastuzumab: one patient had trastuzumab with weekly vinorelbine, 60 mg/m^2^, with no adverse effect, whereas the treatment of the other two patients was associated with the development of anhydramnios, which subsided after treatment withdrawal [42].

Hormonal Agents

Tamoxifen has been shown to be potentially teratogenic in animal studies. Although tamoxifen has safely been given in pregnant patients with metastatic disease without damage to the child, there are reports of birth defects, such as ambiguous genitalia and craniofacial defects. Therefore, the use of tamoxifen is usually delayed until the end of pregnancy. The long-term effects of tamoxifen use and whether it may increase gynecological cancers in daughters (as diethylstilbestrol does) are unknown; in pregnant rats, tamoxifen has been associated with breast cancer in the female offspring. Per updated guidelines, tamoxifen and trastuzumab are contraindicated owing to their reported fatal fetal effects.

Bisphosphonates

The use of pamidronate was reported in three patients with malignancy-associated hypercalcemia. Two neonates developed transient hypocalcemia but without any developmental problem [40]. It is possible that this hypocalcemia was a result of parathyroid suppression in the neonates due to maternal hypercalcemia, rather than a direct effect of pamidronate on the fetus. Hence, it is recommended that when bisphosphonates are used in pregnant women, the neonatal calcium levels should be carefully monitored.

Termination of pregnancy

Consideration of pregnancy termination depends on the prognosis of cancer and the treatment options. Termination of pregnancy may allow earlier adjuvant radiotherapy but does not seem to improve prognosis or survival [43]. Even in the advanced stages, elective abortion in combination with castration (bilateral oophorectomy) to decrease the potential hormonal stimulation of breast cancers has not been shown to improve survival.

Prognosis

Pregnancy Before Breast Cancer

Some studies have found that the prognosis in women who were diagnosed as having breast cancer within two years of giving birth was poorer than that in other women with breast cancer; however, other studies did not observe this difference [44-45]. More research is needed in this area.

Pregnancy During Breast Cancer

Despite the responsiveness of breast tissue to hormonal stimulation in the pregnant and lactation state (when controlled for patient age and disease stage), the prognosis does not seem to differ from that in non-pregnant patients of the same age and stage of disease [46]. There is a school of thought among doctors that terminating the pregnancy may help slow the course of more advanced breast cancers; however, conducting research in this area is difficult and very few quality studies have been performed. The studies conducted to date have not shown that terminating the pregnancy improves a woman's prognosis. Moreover, studies have not demonstrated that the types of treatment delays that are sometimes needed during pregnancy have an effect on breast cancer outcome; however, again, this has proven to be a difficult area to study.

Pregnancy After Breast Cancer Treatment

Certain chemotherapeutic drugs may affect a woman’s fertility, but many women will still be able to become pregnant after treatment. The percentage of patients who have full-term pregnancies after a breast cancer diagnosis is minuscule. Among women younger than 45 years at diagnosis, only 3% subsequently give birth to a live infant, and among women younger than 35 years at diagnosis, 8% have full-term pregnancies [47]. Various factors may contribute to the low pregnancy rate, and among them is the increased chance of spontaneous abortions, occurring in nearly 25% of pregnancies [47]. It is uncertain whether women with a history of breast cancer have an increased risk of cancer recurrence after becoming pregnant. Most of the previous studies found that pregnancy does not increase the risk of recurrence after successful treatment of breast cancer. The link between estrogen levels and growth of breast cancer cells is well established, and because of this link, the general advice for breast cancer survivors is to wait at least two years before attempting to become pregnant. This would allow any early relapse to be diagnosed, which could affect a woman’s decision to become pregnant in the future. Moreover, this advice is not based on evidence from clinical trials, and the decision is influenced by many factors, including the woman’s desire for further pregnancies, age, statistical risk of an early relapse from cancer, and potential impact of estrogens on the risk of breast cancer recurrence.

Hormonal therapeutic drugs, such as tamoxifen, could affect a developing fetus and should be avoided. There is no evidence that a previous history of breast cancer in the mother has any effect on the baby. The survival of pregnant and non-pregnant patients with breast cancer has been reported to be equal. The prognosis does not improve for pregnant patients who aborted and received standard treatment for the disease.

Multidisciplinary approach

Genetic counseling is recommended for all pregnant patients with breast cancer, as one case-control study showed that a family history of breast cancer was three times more common in pregnant and lactating women than in non-pregnant women with breast cancer [48]. Further studies are needed to explore the potential relationship between mutations of individual breast cancer susceptibility genes and the subsequent development of breast cancer during pregnancy. In carriers of breast cancer susceptibility genes 1 and 2 (BRCA1/BRCA2) mutations, pregnancy also increases the risk of breast cancer. Cullinane et al. showed that in BRCA1 carriers, the risk of breast cancer decreases significantly after four or more births, whereas in BRCA2 carriers, parity after two or more births increases the risk of breast cancer (by 1.5-fold compared to nulliparous women) [49]. In BRCA2 mutation carriers, the breast cancer risk within two years after delivery is 70% higher than that in nulliparous women. Cancer during pregnancy is a stressful situation; therefore, measures should be taken to provide psychological support during treatment and delivery to the patient and her family.

Monitoring of pregnancy

Before starting chemotherapy, fetal ultrasonography should be performed to ensure that the fetus appears normal, and the gestational age and expected date of delivery should be estimated. Ultrasonography should be performed before every cycle of chemotherapy to assess fetal growth. Any fetal abnormalities and pregnancy-related complications should be treated based on the standard recommendations.

Delivery and postpartum

Studies have reported that women with hematological and non-hematological malignancies who received chemotherapy are more likely to deliver prematurely. When fetal maturation is sufficient, labor can be induced or a cesarean section can be performed in a hospital with adequate neonatal support. The timing of the delivery can be optimized with the treatment of the mother’s breast cancer. It is recommended that the timing of delivery be approximately three weeks after the last dose of chemotherapy [39]. The placenta should be sent for histopathological examination to rule out the rare possibility of placental metastases.

Breastfeeding

Breastfeeding during chemotherapy and hormonal therapy is contraindicated, as most drugs can be excreted in breast milk. Nevertheless, there is no evidence to support the belief that women who have completed treatment for breast cancer cannot breastfeed safely from the unaffected breast. One study showed that bilateral lactation could be possible after conservative surgery and radiation therapy in approximately 25% of women [50].

Future pregnancy

Women should be advised that premature menopause might result from breast cancer treatments, especially if chemotherapy is given to patients older than 30 years. It is recommended that pregnancy should be delayed for at least two years after treatment completion. The recommendation for future conception may be based on the prognosis of individual women. Women with Stage IV disease should not consider pregnancy, and women with Stage III disease should avoid becoming pregnant for at least five years after treatment. Women with recurrent Stage I or II tumors should not consider conception because of the intensity of the required treatment and the poor prognosis.

## Conclusions

As more women postpone childbearing until middle age, the incidence of breast cancer in pregnancy is increasing and delays in diagnosis are more common. It is emphasized that women should undergo periodic breast examinations during prenatal visits. Among the investigations, mammography is of limited value because of pregnancy-induced changes in the breast and some amount of associated risk to the developing fetus in the later part of pregnancy. Fine-needle aspiration or core needle biopsy has emerged as the diagnostic tool of choice in pregnant women with breast cancer. Furthermore, ultrasonography is also a useful diagnostic tool, along with a clinical examination.

Regarding the treatment options, depending on the time of diagnosis, mastectomy and axillary dissection are the traditional treatments of choice. Lumpectomy followed by irradiation can be done after delivery. Radiation during pregnancy is best avoided to prevent the risk to the fetus. In addition to their long-term effects, chemotherapy and hormonal therapy have largely been proven to be safe after the second trimester and in the third trimester.

We recommend providing genetic counseling to all patients with pregnancy-associated breast cancer to serve as a guide for future pregnancies.
